# Linguistic and cultural adaptation to the Portuguese language of antimicrobial dose adjustment software

**DOI:** 10.31744/einstein_journal/2020AO5023

**Published:** 2020-01-23

**Authors:** Samuel Dutra da Silva, Geisa Cristina da Silva Alves, Farah Maria Drumond Chequer, Andras Farkas, Gergely Daróczi, Jason A. Roberts, Cristina Sanches

**Affiliations:** 1 Universidade Federal de São João del-ReiDivinópolisMGBrazilUniversidade Federal de São João del-Rei, Divinópolis, MG, Brazil.; 2 Universidade de ItaúnaItaúnaMGBrazilUniversidade de Itaúna, Itaúna, MG, Brazil.; 3 Optimum Dosing StrategiesBloomingdaleNJUnited StatesOptimum Dosing Strategies, Bloomingdale, NJ, United States.; 4 University of QueenslandQueenslandQLDAustraliaUniversity of Queensland, Queensland, QLD, Australia.

**Keywords:** Software, Dosage forms, Anti-infective agents, Piperacillin, Intensive care units, Surveys and questionnaires, Brazil

## Abstract

**Objective:**

To adapt an antibiotic dose adjustment software initially developed in English, to Portuguese and to the Brazilian context.

**Methods:**

This was an observational, descriptive study in which the Delphi method was used to establish consensus among specialists from different health areas, with questions addressing the visual and operational aspects of the software. In a second stage, a pilot experimental study was performed with the random comparison of patients for evaluation and adaptation of the software in the real environment of an intensive care unit, where it was compared between patients who used the standardized dose of piperacillin/tazobactam, and those who used an individualized dose adjusted through the software Individually Designed and Optimized Dosing Strategies.

**Results:**

Twelve professionals participated in the first round, whose suggestions were forwarded to the software developer for adjustments, and subsequently submitted to the second round. Eight specialists participated in the second round. Indexes of 80% and 90% of concordance were obtained between the judges, characterizing uniformity in the suggestions. Thus, there was modification in the layout of the software for linguistic and cultural adequacy, minimizing errors of understanding and contradictions. In the second stage, 21 patients were included, and there were no differences between doses of piperacillin in the standard dose and adjusted dose Groups.

**Conclusion:**

The adapted version of the software is safe and reliable for its use in Brazil.

## INTRODUCTION

Bacterial infections are an important cause of morbidity and mortality, and are among the ten major causes of death in the world population, especially in low income countries.^1^ Additionally, bacterial resistance to antibiotics is a global concern and a great problem for public health worldwide. In March 2015, the World Health Organization (WHO) proposed a plan of action to combat bacterial resistance, considering that adequate prescription and administration should be considered priority for reducing the growing resistance to antibiotics, and rational and responsible use was one of the actions suggested.^2,3^

The use of standardized doses of antibiotics is a modifiable risk factor for emergency of bacterial resistance that most healthcare professionals have ignored. Adjustment and individualization of dose, by means of methods employing the pharmacokinetic/pharmacodynamic (PK/PD) correlation, are tools with potential to improve clinical prognoses in some scenarios, as well as to assist reducing the incidence of resistance, since they allow individualization of dose, thus reaching therapeutic concentrations of the drug.^4,5^

Currently, there are software developed in North America and Europe that allow the individualization and dose adjustment, based on the use of populations’ PK.^6^ None of these software was adapted for the Portuguese language, nor validated for the Brazilian population.

The contents should not be translated only linguistically, but should also be culturally adapted to maintain validity and understanding in different cultures. Transcultural adaptation seeks to gain equivalence of content, enabling validity and reliability of information.^7^

The use of applications for adjustment of antibiotic dose may improve the treatment of bacterial infections, and for their correct use, there is need for a clear, precise, and Portuguese software for its use in Brazil.

## OBJECTIVE

To adjust and adapt a software for dose adjustment of antibiotics, initially prepared in English, to Portuguese for use with the Brazilian population; to evaluate the software adaptation in the real setting of an intensive therapy unit.

## METHODS

This is an observational and descriptive study conducted during the period from December 4, 2016 to December 4, 2017. During the first phase for linguistic and cultural adaptation of the instrument software, the Delphi technique was used.

The software used in this present study is the Individually Designed Optimum Dosing Strategies (ID-ODS) 2014 (http://www.optimum-dosing-strategies.org), an application available via internet, which has the objective of obtaining individually optimized antibiotic doses. It uses methods based on statistical models and Monte Carlo simulation, with information arising from certain patient populations, and the objective of providing the maximum chance of positive clinical results. It is a tool with simulation resources available, and an extensive library constructed from population pharmacokinetic models.

A total of 128 specialists in pharmacy, infectious diseases, internal medicine, intensive care medicine, and researchers were invited to participate as judges of the study. These professionals came from various healthcare organizations, chosen from a list of e-mails of professionals involved with intensive care, at organizations with intensive care units, at database of the researchers’ personal e-mails, in addition to the e-mails of professionals involved with pharmacology research.

**Figure 1:**
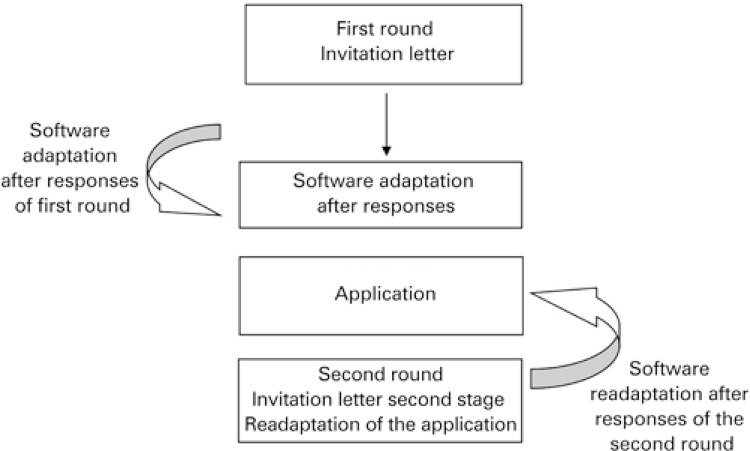
Stages of the Delphi technique for evaluation of Individually Designed Optimum Dosing Strategies software

The investigation tool was developed by means of the Google Forms platform, which consisted of an invitation letter for acceptance or rejection, followed by a questionnaire to be completed, with some of the questions classified as yes or no, and questions about the level of agreement with alternatives from zero to 10. In the first stage, an invitation letter was sent by e-mail to the judges. The letter was again sent by e-mail to remind the non-respondents at the 7^th^, 15^th^, and 30^th^ day after the first invitation. The participants who did not fill out the questionnaire after the three attempts were considered as refusals and were excluded from the study. The e-mails that returned, after verification of their correct typing, were excluded. Rounds were made (Figure 1), until attaining consensus defined as agreement superior to 80%. During the first round, consensus superior to 80% was obtained; the second round only performed the modifications suggested by the first round participants, and once again the level of agreement was checked after these modifications.^8-11^ For the other rounds, following the criteria of the first round, we invited all the judges who responded to the first questionnaire.

Questions were asked that cover the visual and operational aspects of the computer program, which is visual presentation of the software; clarity and precision of information; “Do you consider the platform easy to access?”; “Is any information dubious?”; “Is the time necessary to wait for the graph and dose information of the antibiotic to be generated acceptable?”; “Is the graph easy to understand?”; “Would you change any information on the graph?”; “Would you use this application in your daily clinical practice?”; “What score would you give for this application?”; and “Would you have any suggestion or criticism for the improvement of the application?”.

In the second stage, a pilot was conducted to assess the adaptation of the software in a real environment of an intensive care unit (ICU). An experimental, prospective study was done, with random allocation, in which the differences among the doses of piperacillin/tazobactam, and variables of interest of individuals who receive the usual standardized dose of piperacillin/tazobactam, and those who took the adjusted individualized dose by means of the ID-ODS. The study was developed at the ICU of a medium-sized hospital in the Midwest region of the state of Minas Gerais. The ICU had ten beds and was of “size II,” attending the adult population. Piperacillin/tazobactam was the study drug, since this had been recently implanted into the protocol of antimicrobials of the organization, and limited for use only in the ICU patients, facilitating randomization and dose adjustment, whenever necessary, for each patient.

All patients admitted to the ICU who received piperacillin/tazobactam within the period of 12 months: from February 1^st^, 2017 to February 1^st^, 2018 were recruited. Patients aged over 18 years; confirmed or suspected infection; indications for use of the piperacillin/tazobactam antibiotic, as per institutional protocol were eligible. Excluded were pregnant women; individuals positive for human immunodeficiency virus (HIV) or hepatitis B or C virus; with allergy to the antibiotic utilized; patients previously enrolled in this study; with creatinine >2mg/dL, or elevation superior to twice the baseline value; with insufficient data to calculate the initial dose by means of the software.

The variables of interest evaluated, obtained by means of patient medical records, were global mortality in 30 days; length of stay at the ICU; number of days on antibiotic; serum creatinine >2mg/dL or increase by two-fold within the previous 72 hours; variation of creatinine upon admission and after 72 hours; score on the Simplified Acute Physiology Score (SAPS 3), of the Multiple Organ Dysfunction Score (MODS), and Sequential Organ Failure Assessment (SOFA) at the time of randomization; score for MODS and SOFA on days 5, 7, and 14 of treatment with the piperacillin+tazobactam; and score on SAPS 3, on day 28 of treatment.

Oxygen pressure (PaO_2_), inspired oxygen fraction (FiO_2_), platelets, bilirubin, creatinine, heart rate, central venous pressure, mean arterial pressure, total leukocyte count, axillary temperature, oxygen saturation (SatO_2_), bicarbonate, and Glasgow coma scale, were the data needed for the calculation of SAPS 3, SOFA, and MODS.

For the software dose adjustment, we used age, height, weight, sex, creatinine, site of patient hospitalization, and minimum inhibitory concentration (MIC).

Allocations of patients were obtained by means of arbitrary numbers randomized in block obtained by the Stats Direct software, with the option of the balanced allocation option in blocks of 20. Participants of the clinical study had no knowledge of the group to which they had been allocated.

Two groups were formed; the control with the initial empirical dose calculated by means of the recommendations of the Infection Control team of the organization (tazobactam 4.5g intravenously every 8 hours), and the intervention with an adjustment of the individualized dose, calculated by the antibiotic dosage software, ID-ODS, adapted for Portuguese, and available on a computer.

Prolonged infusion doses were not used during the research period at the ICU. The duration of treatment followed the internal protocol for the administration of antimicrobials of 10 to 14 days.

The software was presented to all prescribers of the ICU, with training simulating individual situations for obtaining the doses and orientation of the prescriber as to use of the software. Meetings were held with the technical pharmacy and nursing teams in order to clarify any doubts related to fractioning, administration, and storage of the doses, to guarantee patient safety. The technical team was also instructed to report any adverse event related to the drug.

A descriptive analysis was done of the data by means of distribution of frequency and measurements of central tendency. The categorical variables (sex, sepsis, and prior use of an antibiotic) were expressed as frequency and percentage, and analyzed using Fisher’s exact test and Pearson’s χ^2^ test. For numerical variables, first the normality of the data was verified, and then the analyses of comparison. For data that did not present with normal distribution (age, weight, height, SOFA, MODS, SAPS, daily dose, length of stay at ICU, serum creatinine, and dose administered with the interval of doses), the Mann-Whitney test was used. All analyses were performed considering significance level of 5%, conducted with the (SPSS) software, version 19.

The study was approved by the Ethics Research Committee Involving Human Beings, of the *Universidade Federal de São João Del Rei* (UFSJ), Central-West Campus, approval protocol no. 1.835.004, CAAE: 56916216.5.0000.5545, on November 14, 2016. All participants or their legal guardians were instructed and invited to sign the Informed Consent Form (ICF).

## RESULTS

**Figure 2:**
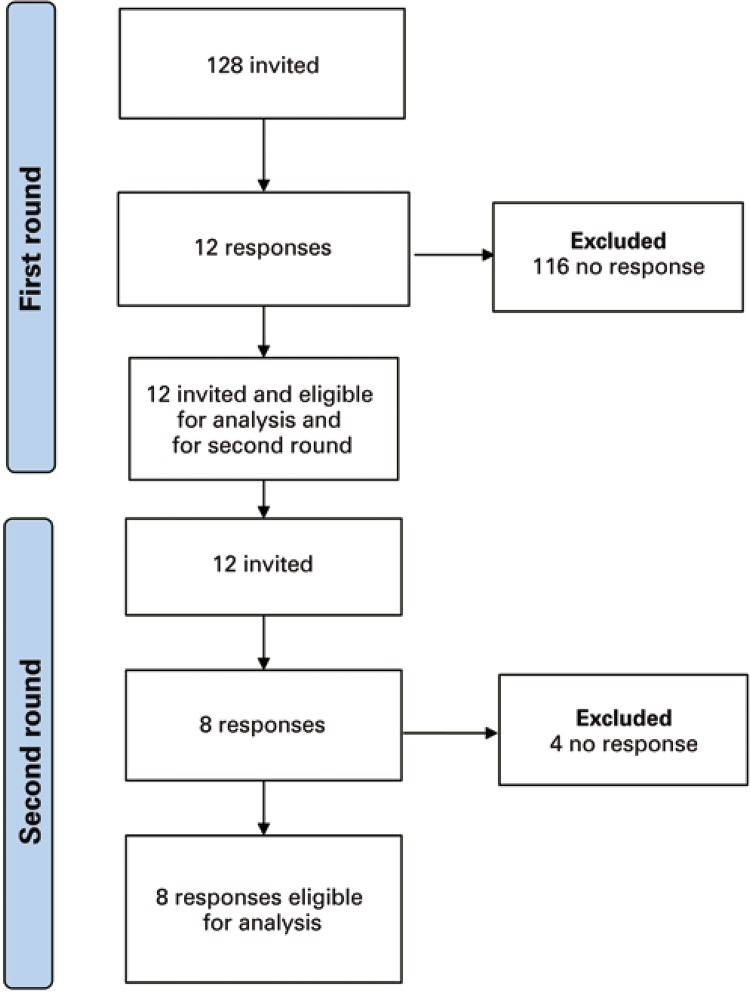
Flowchart of answers from the first and second rounds

A total of 128 invitations were sent to the judges, and seven returned from the mailbox, with 121 duly sent. Of these, 12 responded, as shown in figure 2.

Six physicians (three infectious disease specialists and three intensive care specialists), four pharmacists who are university lecturers and researchers, and two health science graduate students (one master’s and one doctorate degree) participated in the first round, as displayed in table 1.

**Table 1:** Profile of professionals who responded to the Delphi questionnaire

Professionals	First round	Second round
Physicians	6 (50)	4 (50)
Pharmacists	4 (33)	3 (37.5)
Researchers (Master’s and doctorate students)	2 (17)	1 (12.5)
Total	12 (100)	8 (100)

Results expressed as n (%).

The consensus obtained for the first round was superior to 80%, and the suggestions derived from the judges answers were forwarded to the developer of the software, for adaptation and adjustments. Later, they were submitted to the second round for verification and permanence of agreement. In the second round, the judges determined a consensus superior to 90% of the questions, as per table 2.

**Table 2:** Consensus of the judges using the Delphi technique in the first (n=12) and second rounds (n=8)

Consensus	First round	Second round
Visual presentation of the software?	8.9±0.83	9.4±0.74
Is the information clear and precise?	9.0±0.89	9.1±0.64
Is the platform easy to access?	9.1±1.14	9.4±0.52
What score would you give to this application?	8.58±1.16	9.25±0.89

Results expressed as mean±standard deviation. Scores attributed from 1 to 10: The lower the score, the higher the score the better.

The frequency of the main suggestions obtained by the judges, important for the adaptation of the software, is shown on table 3.

**Table 3:** Suggestions and considerations of adaptation of the Individually Designed-Optimum Dosing Strategies software

Questions	Responses	First round	Second round
Would you modify any information on the graph?	No	7 (58.3)	4 (50)
	Improve highlight of the dose, and better distribution/positioning of infusion time and creatinine clearance	2 (16.6)	-
	Improve legends of the graphs (should be self-explanatory)	1 (8.3)	1 (12.5)
Would you have any other suggestion or criticism for the improvement of the application?	Consider the presence of more co-morbidities	1 (8.3)	1 (12.5)
	Adjust and centralize the software layout	2 (16.6)	-
	Clarify which MIC is to be considered	2 (16.6)	2 (25)
	Correct the periods and commas, as these can lead to divergent results when entered	1	-
Is any information dubious?	The value of MIC to be considered is not clear	2	1 (define MIC and what its clinical significance is)
	Initial entry, “more.” Initial entry of the software is dubious.	3	-

Results expressed as n (%). MIC: minimum inhibitory concentration.

Changes were made in the software layout before and after the judges’ suggestions, but no difficulties were recorded as to the use of the software. It was then implemented in the ICU routine, for the performance of the second stage of the research.

In the second stage, ICU admitted, during the recruitment period, 29 patients with indications for use of piperacillin/tazobactam. Twenty-one of these met all the requirements for inclusion in the study, in which 20 patients came from the first randomization block and one from the second block.

The groups were composed of predominantly male patients (66.6%) with ages varying between 48 and 87 years, and the median among the groups was 70.14 years for the dose adjusted group and 67.0 years for the standardized dose group. Table 4 presents the anthropometric characteristics of the groups. There was no statistically significant difference between them in any of the sociodemographic variables evaluated in this study (p>0.05).

**Table 4:** Anthropometric characteristics of the standardized dose and adjusted dose groups

Characteristic	Adjusted dose group (n=9)	Standardized dose group (n=12)	p value
Age, years	70.14 (48-87)	67.0 (57.0-79.0)	0.367^*^
Sex			
Female	4 (44.4)	3 (25.0)	0.397^†^
Male	5 (55.6)	9 (75.0)	
Height, meter	1.63 (1.57-1.67)	1.62 (1.56-1.68)	0.968^*^
Weight, kg	65.2 (58.6-97.6)	75.2 (60.9-92.3)	0.604^*^

Results expressed as median and interquartile range or n (%).

^*^ Mann-Whitney test; ^†^ Fisher’s exact test.

Results of the cultures of biological material were available for 18 (86%) patients. There was no growth detected of bacteria in the cultures of nine patients (43%), and for the patients with results presenting bacterial growth, eight (38%) presented with a susceptibility profile of ‘sensitive’, and one (4.8%) as ‘intermediate’ to piperacillin/tazobactam. For the other patients, no cultures were ordered (3; 14.3%).

No differences were noted for the total doses administered between the groups. On the other hand, both the dose administered by the hour and the frequency of administration of these doses showed a difference between the groups (p<0.0001). As to the clinical profile of the study participants, there was no difference relative to the clinical and laboratory variables at the beginning of antibiotic treatment (D0), as presented on table 5.

**Table 5:** Clinical and laboratory characteristics and dose administered of piperacillin/tazobactam in the standardized dose and dose adjusted groups at the beginning of antibiotic treatment (D0)

Variable	Adjusted dose group (n=9)	Standardized dose group (n=12)	p value
Sepsis, n (%)			
Yes	5 (55.6)	8 (66.7)	0.673^*^
No	4 (44.4)	4 (33.3)	
Prior use of antibiotic, n (%)			
Yes	8 (88.9)	8 (66.7)	0.338^*^
No	1 (11.1)	4 (33.3)	
Source of infection			
Community-acquired pneumonia, n (%)	3 (33.3)	2 (16.7)	0.969^†^
Healthcare-associated pneumonia	5 (55.6)	8 (66.6)	
Others, n (%)	1 (11.1)	2 (16.7)	
SAPS 3	63.0 (51.0-69.5)	51.5 (43.3-63.5)	0.193^‡^
SOFA	5.0 (4.5-6.0)	5.5 (3.3-7.0)	0.862^‡^
MODS	3.0 (1.5-3.5)	2.5 (2.0-4.0)	0.862^‡^
Daily dose, g	9.45 (9.0-19.63)	13.5	0.4221^‡^
Frequency of administration, n (%)			
3 times (every 8 hours)	0 (0)	12 (100)	<0.0001
4 times (every 6 hours)	5 (55.6)	0 (0)	
6 times (every 4 hours)	4 (44.9)	0 (0)	
Dose administered in the dose interval, g	2.25 (2.25-3.28)	4.5	<0.0001
Serum creatinine, mg/dL	0.9 (0.6-1.1)	1.1 (0.9-1.6)	0.129

Results expressed by median and interquartile range or n (%).

^*^ Fisher’s exact test; ^†^ Pearson χ^2^ test; ^‡^ Mann-Whitney test.

SAPS 3: Simplified Acute Physiology Score; SOFA: Sequential Organ Failure Assessment; MODS: Multiple Organ Dysfunction Score.

When evaluating the scores and outcomes, an absence of statistically significant differences was noted, except for MODS on the fifth day of antibiotic treatment (D5). It is noteworthy that despite the standardized dose group having lower values of MODS on D5, this difference was not maintained throughout treatment. The calculation of SAPS 3 on the 28^th^ day was not possible, since there were few patients for analysis (Table 6).

**Table 6:** Clinical and laboratorial outcomes of the standardized dose and adjusted dose groups during treatment (D3, D14, D30)

Variable	Adjusted dose group (n=9)	Standardized dose group (n=12)	p value
Length of stay at ICU, days	17.0 (14.0-22.0)	8.5 (6.3-17.5)	0.219^*^
Days at ICU, n (%)			
≤14	1 (11.1)	5 (41.7)	0.148^†^
>14	8 (88.9)	7 (58.3)	0.382^†^
Days of antibiotic	7.0 (7.0-10.0)	6.3 (6.0-7.3)	0.277
Serum creatinine on D3, mg/dL	0.9 (0.6-1.3)	0.9 (0.8-1.5)	0.837^*^
SOFA			
D0	5.2 (3-8)	5.4 (1-9)	0.382^*^
D5	6.0 (3.5-6.0)	3.5 (1.0-6.8)	0.601^*^
D7	3.5 (1.8-6.3)	4.0 (3.0-6.0)	1.0^*^
D14	3.5 (1.8-4.8)	3.0 (2.0-4.5)	0.827^*^
MODS			
D0	2.6 (0-4)	2.8 (1-5)	0.049^*^
D5	3.0 (1.5-4.5)	1.0 (0.3-2.8)	0.669^*^
D7	1.5 (0.8-5.0)	2.0 (1.5-4.0)	0.329^*^
D14	1.5 (0.8-3.0)	3.0 (1.5-3.0)	0.382^†^
Progression^‡^			
End of treatment, n (%)	6 (75)	5 (41.7)	0.197^†^
Death, n (%)	2 (25)	7 (58.3)	

Results expressed by median and interquartile range or n (%).

* Mann-Whitney test; ^†^ Fisher’s exact test; ^‡^ n=20; one of the participants presented with a culture result showing intermediate resistance to piperacillin+tazobactam, and the antibiotic treatment was modified.

ICU: intensive care unit; SOFA: Sequential Organ Failure Assessment; MODS: Multiple Organ Dysfunction Score.

There was good acceptance by the team of prescribers as to the use of the software. No adverse events were recorded related to the use of piperacillin/tazobactam, but there were several records of the nursing team as to difficulties found in the administration of piperacillin/tazobactam during the study period.

## DISCUSSION

The use of antibiotic dose adjustment and individualization software may contribute to the rational use of antibiotics, optimizing and maximizing its therapeutic efficacy.^6^ On the other hand, its use in a foreign language, with no linguistic and cultural adaptation for the Portuguese language, can trigger errors in understanding and lead to contradictions.^7^ Thus, its adaptation and validation are means of avoiding possible errors or misunderstandings related to linguistic setting, as was verified in the present study – for example, adjustments in language, such as replacing periods by commas, were needed so that no diversions were caused in the calculations of the software.

There was agreement superior to 80% in all items evaluated in the first round, and after adjustments, in the second round, an increase in agreement was noted. Reaching an agreement superior to 90% in all questions showed homogeneity in the judges’ assessment, despite the different areas of expertise and professions.

The suggestion of some of the judges who requested a better characterization of the MIC is also noteworthy, since it is of great importance in bacterial infection control. The understanding of the MIC and of its consequences is of vital importance for an optimal use of the software.

A recent study evaluating the capacity to reach the therapeutic goal in septic and critically ill patients, analyzed the data of 68 ICU in 10 countries and verified that the standardized dose, associated with the habitual administration, did not reach concentrations that were effectively capable of covering all the susceptible organisms.^12^ In other studies, the adjusted dose increased the probability of reaching the recommended therapeutic goals, especially in subpopulations of critically ill patients.^5^The present study demonstrates that using the software for individualized adjustment, and with the MIC goal of 8mg/dL, the total daily doses were statistically similar to those of the standardized dose group. Despite having provided similar total values, the software optimizes the form of administration of the drug, leading to a fractioning of the dose and increasing its frequency of administration.

Several studies have proposed that antimicrobials administered by continuous or prolonged infusion present superior rates of clinical cure when compared to the administration by conventional dose, and that dose individualization based on the Bayesian estimation and on clinical covariables, presents a greater probability of reaching therapeutic concentrations capable of eliminating the infection and reducing the appearance of resistance.^13-16^The present study demonstrates a tendency of lower mortality in the adjusted dose group. Even on the fifth day, when we found higher severity scores in the group with the adjusted dose, as compared to the standardized dose group, we noted a similar and numerically inferior outcome relative to the total number of deaths throughout the study. The low number of participants may have influenced the outcome, precluding extrapolations, but even so, our results allow inferring the possibility of utilizing the dose adjustment software developed based on foreign populations in the Brazilian population.

Elevated doses are often necessary to reach adequate concentrations, to treat infections caused by less susceptible organisms (*e.g.*, *Pseudomonas aeruginosa* and *Acinetobacter baumannii*).^17^Additionally, for time-dependent antibiotics, when the frequency of administration is increased, that is, the interval between doses is reduced, the probability of reaching the PK/PD goal increases considerably, since the time that the antimicrobial needs to be higher than the MIC is reduced. Consequently, the increased percentage of time above the MIC (%T≥MIC) is related to more favorable clinical results.^6,18,19^

One important consideration is that in the present study, the team found it difficult to adjust to fractioning, storage, and administration of piperacillin/tazobactam in fractioned doses and at intervals different from the habitual intervals of the standardized dose. Errors in administration can account for 13% of adverse events at the ICU. Further, potentially avoidable drug-related adverse event rates were two-fold higher at ICU, in comparison with non-intensive care units.^20,21^ In this way, the organizations that opt to utilize adjusted dose need to invest in training of the team and creating safety barriers to avoid such errors.^22^

Within the limitations of the present study, the low number of participants’ responses was noted on the first round. There is no consensus determining the ideal number of participants for the performance of the Delphi technique. Reid^23^observed a variation from 10 to 1,685 participants among the studies. In the present study, the return rate was 10%, that is, a value below those found in online inquiries conducted in North America, Europe, and Oceania, that had a mean of 33%.^24^ Strategies described to increase the response rate, such as resending the questionnaire three times and sending during office hours were used in this study. Some factors may have influenced this result: the participants were invited only by means of an e-mail invitation letter, with no prior personal approach on the part of the researchers; there was no guarantee of reading of the e-mail; and many e-mails may have automatically fallen on the list of spams. Usually, for the statistical analyses, the number of participants is determined after acceptance to participate in the Delphi technique, and the only abstentions counted are those among whom the questionnaire was accepted, but not answered. In our study, all the judges invited whose e-mails did not return were counted for the definition of the response rate, which could explain the divergence with the literature on the first round. The second round, on the other hand, presented a return rate of 72.7%, which is compatible with the literature.^25^

Use of the software translated and adapted for Portuguese and to the Brazilian context occurred with no difficulties. Relative to the treatment of patients with adjusted and standardized doses, no statistical differences were noted between the groups, but more randomized clinical studies are needed to evaluate clinical outcomes among the doses, and the verification of their serum concentrations.

## CONCLUSION

The present study demonstrated that the version of the software in Portuguese is semantically safe and presents with a good level of validity, allowing its reliable use in Brazil as to linguistic and cultural adaptation. Additionally, the conduction of a pilot study allowed us to carry out the adjustments to improve the cultural adaptation of the Individually Designed Optimum Dosing Strategies software and, based on the results obtained, we concluded that its use in critical patients in Brazil is viable. There should be continuity in the evaluation and adjustments of the computer program, until samples of plasma concentrations from the native population base are obtained. Other important points to be considered are the training and orientation of the unit team, for adjustments and appropriate administration of the adjusted doses.
