# Supplementary material for: Linguistic and cultural adaptation to the Portuguese language of antimicrobial dose adjustment software
Source: Einstein (Sao Paulo). 2020 Jan 23;18:eAO5023. doi: 10.31744/einstein_journal/2020AO5023 (PMC6986457; doi:10.31744/einstein_journal/2020AO5023)
Supplement: Translation

# Translation: Adaptação linguística e cultural para a língua portuguesa de um software para ajuste de dose de antimicrobianos

**Authors:** Samuel Dutra da Silva, Geisa Cristina da Silva Alves, Farah Maria Drumond Chequer, Andras Farkas, Gergely Daróczi, Jason A. Roberts, Cristina Sanches

**Affiliations:** 1 Brasil Universidade Federal de São João del-Rei, Divinópolis, MG, Brasil.; 2 Brasil Universidade de Itaúna, Itaúna, MG, Brasil.; 3 United States Optimum Dosing Strategies, Bloomingdale, NJ, United States.; 4 Australia University of Queensland, Queensland, QLD, Australia.

**Keywords:** Software, Formas de dosagem, Anti-infecciosos, Piperacilina, Unidades de terapia intensiva, Inquéritos e questionários, Brasil

## Abstract

**Objetivo:**

Adaptar um software de ajuste de dose de antibióticos inicialmente elaborado em língua inglesa para o português e a conjuntura brasileira.

**Métodos:**

Trata-se de estudo observacional, descritivo, em que foi utilizado o método Delphi para estabelecer consenso entre especialistas de diferentes áreas da saúde, com perguntas que abordaram os aspectos visuais e operacionais do software. Em uma segunda etapa, foi realizado um estudo piloto, experimental, com alocação aleatória dos pacientes, para avaliação e adaptação do software em ambiente real de uma unidade de tratamento intensivo, onde foram comparadas diferenças entre pacientes que utilizaram dose padronizada usual de piperacilina/tazobactam, e os que utilizaram a dose individualizada ajustada por meio do software *Individually Designed Optimum Dosing Strategies*.

**Resultados:**

Participaram da primeira rodada 12 profissionais cujas sugestões foram encaminhadas ao desenvolvedor do software para adequação e ajustes, e posteriormente submetidas à segunda rodada. Oito especialistas participaram da segunda rodada. Foram obtidos índices de 80% e 90% de concordância entre os juízes, caracterizando uniformidade nas sugestões. Dessa forma, houve modificação no layout do software para adequação linguística e cultural, minimizando erros de entendimento e contradições. Na segunda etapa, foram incluídos 21 pacientes, e não houve diferenças entre doses de piperacilina nos grupos dose padronizada e dose ajustada.

**Conclusão:**

A versão adaptada do software é segura e confiável para seu uso no Brasil.

## INTRODUÇÃO

As infecções bacterianas são importante causa de morbimortalidade e estão entre as dez causas de morte da população mundial, principalmente em países de baixa renda.^1^ Adicionalmente, a resistência bacteriana aos antibióticos constitui preocupação global e grande problema de saúde pública mundial. Em março de 2015, a Organização Mundial da Saúde (OMS) propôs um plano de ação para combate à resistência bacteriana, considerando que a prescrição e a administração adequada devem ser consideradas prioridades para a redução da resistência crescente aos antibióticos, e o uso racional e responsável dos antibióticos foi umas das ações sugeridas.^2,3^

O uso de doses padronizadas de antibióticos é fator de risco modificável para a emergência de resistência bacteriana que a maioria dos profissionais de saúde tem ignorado. O ajuste e a individualização de dose, por meio da utilização de métodos que utilizam a correlação farmacocinética/farmacodinâmica (PK/PD), são ferramentas com potencial para melhora dos prognósticos clínicos em alguns cenários e também para auxiliar na redução da incidência de resistência, por permitirem a individualização de dose e, desta maneira, atingir concentrações terapêuticas do fármaco.^4,5^

Atualmente, existem programas computacionais desenvolvidos na América do Norte e Europa que permitem a individualização e o ajuste de dose, baseados na utilização de dados de PK populacional.^6^ Nenhum destes programas foi adaptado para a língua portuguesa e nem validado para a população brasileira.

Os conteúdos não devem ser traduzidos apenas linguisticamente, mas também devem ser adaptados culturalmente, para manter a validade e o entendimento em diferentes culturas. A adaptação transcultural busca conseguir a equivalência do conteúdo, permitindo a validade e a confiabilidade nas informações.^7^

O uso de aplicativos para ajuste de dose de antibióticos pode melhorar o tratamento das infecções bacterianas e, para correta utilização, é necessário um software claro, preciso e no idioma português, para sua utilização no Brasil.

## OBJETIVO

Adequar e adaptar um software de ajuste de dose de antibióticos, inicialmente elaborado em língua inglesa, para o português e para a realidade brasileira, e avaliar adaptação do software em ambiente real de uma unidade de terapia intensiva.

## MÉTODOS

Trata-se de estudo observacional, descritivo conduzido no período de 4 de dezembro de 2016 a 4 de dezembro de 2017. Na primeira etapa para adaptação linguística e cultural do instrumento software, foi utilizada a técnica de Delphi.

O software utilizado no presente estudo é o *Individually Designed Optimum Dosing Strategies* (ID-ODS) 2014 (http://www.optimum-dosing-strategies.org), um aplicativo disponibilizado via internet, que tem como objetivo a obtenção de doses de antibióticos otimizadas individualmente. Utiliza-se de métodos baseados em modelos estatísticos e simulação de Monte Carlo, com informações oriundas de determinadas populações de pacientes, objetivando proporcionar a máxima chance de resultados clínicos positivos. É uma ferramenta com recursos de simulação disponíveis, e extensa biblioteca construída a partir de modelos farmacocinéticos populacionais.

Foram convidados a participar e serem os juízes do estudo 128 especialistas, profissionais de farmácia, infectologia, clínica médica, medicina intensiva e pesquisadores, de diferentes instituições de saúde, escolhidos a partir de uma lista de e-mails de profissionais, envolvidos com terapia intensiva, em instituições com unidades de terapia intensiva, em banco de e-mail pessoal dos pesquisadores, além de e-mails de profissionais envolvidos com pesquisas em farmacologia.

O instrumento de pesquisa foi desenvolvido por meio da plataforma *Google Forms*, que consistiu em carta convite com a opção de aceite ou rejeição, seguida de um questionário para preenchimento, com algumas questões classificadas como sim ou não, e para as questões sobre nível de concordância com alternativas de zero a 10. Na primeira etapa, foi enviada carta convite por e-mail aos juízes. A carta foi reenviada por e-mail para relembrar aos não respondentes no 7º, 15º e 30º dias após o primeiro convite. Os participantes que não preencheram o questionário após as três tentativas foram considerados como recusa e excluídos do estudo. Os e-mails que retornaram, após conferência de sua correta digitação, foram excluídos. Foram realizadas rodadas (Figura 1), até obtenção do consenso definido como concordância superior a 80%. Na primeira rodada, foi obtido consenso superior a 80%; a segunda etapa apenas realizou as modificações sugeridas pelos participantes da primeira rodada, e foi novamente checado o nível de concordância após essas modificações.^8-11^ Para as demais rodadas, foram convidados, seguindo os critérios da primeira rodada, todos os juízes que responderam ao primeiro questionário.

**Figura 1:** Etapas da técnica Delphi para avaliação do software* Individually Designed Optimum Dosing Strategies*

Foram realizadas perguntas que abordaram os aspectos visuais e operacionais do programa computacional, a saber: apresentação visual do software; clareza e precisão das informações; “Você considera a plataforma de fácil acesso?”, “Alguma informação é duvidosa?”; “O tempo necessário para aguardar a geração do gráfico e das informações da dose do antibiótico é aceitável?”; “O gráfico é de fácil entendimento?”; “Você modificaria alguma informação no gráfico?”; “Você utilizaria esse aplicativo em sua prática clínica?”; “Que pontuação você daria para este aplicativo?”; e “Você teria mais alguma sugestão ou crítica para o aprimoramento do aplicativo?”.

Já para a segunda etapa, foi realizado um piloto para avaliação da adaptação do software em ambiente real de uma unidade de terapia intensiva (UTI). Foi realizado estudo experimental, prospectivo, com alocação aleatória, tendo sido comparadas as diferenças entre doses de piperacilina/tazobactam, e variáveis de interesse de indivíduos em uso de dose padronizada usual de piperacilina/tazobactam, e os que utilizaram a dose individualizada ajustada por meio do ID-ODS. O estudo foi desenvolvido na UTI de um hospital de médio porte do centro-oeste de Minas Gerais. A UTI possuía dez leitos e era de porte II, atendendo a população adulta. A piperacilina/tazobactam foi o fármaco de pesquisa, uma vez que esta foi implementada no protocolo de antimicrobianos da instituição recentemente, sendo limitado o uso apenas para pacientes da UTI, facilitando a randomização e o ajuste dose, quando necessário, de cada paciente.

Foram recrutados todos os pacientes admitidos na UTI que receberam piperacilina/tazobactam no período de 12 meses: de 1º de fevereiro de 2017 até o dia 1º fevereiro de 2018. Foram elegíveis para participar do estudo os pacientes com idade superior a 18 anos; infecção confirmada ou suspeita; indicação de uso do antibiótico piperacilina/tazobactam, conforme protocolo institucional. Foram excluídos gestantes; indivíduos portadores do vírus da imunodeficiência humana (HIV) ou de hepatite B ou C; com alergia ao antibiótico utilizado; inscritos previamente neste estudo; com creatinina >2mg/dL, ou elevação superior a duas vezes o valor basal; com insuficiência de dados para calcular a dose inicial por meio do software.

As variáveis de interesse avaliadas, obtidas por meio de prontuários de pacientes, foram mortalidade global em 30 dias; tempo de permanência em UTI; número de dias de uso de antibiótico; concentração de creatinina sérica >2mg/dL ou aumento em duas vezes nas últimas 72 horas; variação da creatinina na admissão e após 72 horas; pontuação do *Simplified Acute Physiology Score *(SAPS 3), do *Multiple Organ Dysfunction* Score (MODS) e *Sequential Organ Failure Assessment* (SOFA) no momento da randomização; pontuação MODS e SOFA nos dias 5, 7 e 14 de tratamento com o antibiótico piperacilina+tazobactam; e pontuação SAPS 3, no dia 28 de tratamento.

Pressão de oxigênio (PaO_2_), fração inspirada de oxigênio (FiO_2_), plaquetas, bilirrubinas, creatinina, frequência cardíaca, pressão venosa central, pressão arterial média, global de leucócitos, temperatura axilar, saturação de oxigênio (SatO_2_), bicarbonato e escala de coma de Glasgow foram os dados necessários para cálculo do SAPS 3, do SOFA e do MODS.

Para o ajuste de dose do software, foram usados: idade, altura, peso, sexo, creatinina, local de internação do paciente e concentração inibitória mínima (CIM).

A alocação dos pacientes foi obtida por meio de números aleatórios randomizados em bloco obtidos pelo software *StatsDirect*, na opção alocação balanceada em blocos de 20. Os participantes do ensaio clínico não tiveram conhecimento do grupo em que foram alocados.

Foram formados dois grupos, sendo o controle com dose inicial empírica calculada por meio das recomendações da Comissão de Controle de Infecção Hospitalar da instituição (tazobactam 4,5g por via endovenosa a cada 8 horas) e o intervenção com ajuste de dose individualizada, calculada pelo software de dosagem de antibiótico ID-ODS adaptado para o português, disponível em um computador.

Doses de infusão prolongada não foram utilizadas durante o período de pesquisa na UTI. O tempo de duração de tratamento seguiu o protocolo interno de administração de antimicrobianos de 10 a 14 dias.

Foi apresentado o software para todos os prescritores da UTI, realizando treinamento individual simulando situações para obtenção de doses e orientação do prescritor no uso do software. Foram feitas reuniões com equipe técnica de farmácia e enfermagem, para sanar quaisquer dúvidas em relação a fracionamento, administração e armazenamento das doses, para garantir segurança ao paciente. A equipe técnica também foi orientada a notificar qualquer evento adverso relacionado ao fármaco.

Foi realizada análise descritiva dos dados, por meio da distribuição de frequência e medidas de tendência central. As variáveis categóricas (sexo, sepse e uso prévio de antibiótico) foram expressas em frequência e percentagem, e analisadas utilizando o teste exato de Fisher e teste χ^2^ de Pearson. Para as variáveis numéricas, primeiramente foi verificada a normalidade dos dados e, posteriormente, foram realizada análises de comparação. Para os dados que não apresentam distribuição normal (idade, peso, altura, SOFA, MODS, SAPS, dose diária, tempo de internação UTI, creatinina sérica e dose administrada no intervalo de dose), utilizou-se o teste de Mann-Whitney. Todas as análises foram realizadas considerando o nível de significância de 5%, realizadas pelo programa (SPSS), versão 19.

O estudo foi aprovado pelo Comitê de Ética em Pesquisas Envolvendo Seres Humanos da Universidade Federal de São João Del Rei (UFSJ), Campus Centro-Oeste, protocolo de aprovação 1.835.004, CAAE: 56916216.5.0000.5545 em 14 de novembro de 2016. Todos os participantes ou seus responsáveis legais foram orientados e convidados a assinar o Termo de Consentimento Livre e Esclarecido (TCLE).

## RESULTADOS

Foram enviados 128 convites aos juízes, sete retornaram na caixa de correios, totalizando 121 enviados. Destes, foram obtidos 12 retornos, conforme mostra a figura 2.

**Figura 2:** Fluxograma de respostas da primeira e segunda rodada

Participaram da primeira rodada seis médicos (três infectologistas e três intensivistas), quatro farmacêuticos docentes e pesquisadores e dois pós-graduandos (um mestrando e um doutorando) da área de ciências da saúde, conforme tabela 1.

**Tabela 1:** Perfil dos profissionais que responderam ao questionário Delphi

Profissionais	Primeira rodada	Segunda rodada
Médicos	6 (50)	4 (50)
Farmacêuticos	4 (33)	3 (37,5)
Pesquisadores (mestrandos e doutorandos)	2 (17)	1 (12,5)
Total	12 (100)	8 (100)

Resultados expressos como n (%).

O consenso obtido na primeira rodada foi superior a 80%, e as sugestões derivadas das respostas dos juízes foram encaminhadas ao desenvolvedor do software, para adequação e ajustes. Posteriormente foram submetidas à segunda rodada, para verificação e permanência de concordância. Na segunda rodada, os juízes determinaram um consenso superior a 90% dos questionamentos, conforme tabela 2.

**Tabela 2:** Consenso de juízes pela técnica Delphi na primeira (n=12) e segunda rodadas (n=8)

Consenso	Primeira rodada	Segunda rodada
Apresentação visual do software?	8,9±0,83	9,4±0,74
Informações estão claras e precisas?	9,0±0,89	9,1±0,64
Plataforma de fácil acesso?	9,1±1,14	9,4±0,52
Que pontuação você daria para este aplicativo?	8,58±1,16	9,25±0,89

Resultados expressos como média±desvio padrão. Notas atribuídas de 1 a 10: Quanto menor a pontuação pior, quanto maior a pontuação melhor.

A frequência das principais sugestões obtidas pelos juízes, importantes para a adequação do software, encontra-se na tabela 3.

**Tabela 3:** Sugestões e considerações de adequação do software *Individually Designed-Optimum Dosing Strategies*

Perguntas	Respostas	Primeira rodada	Segunda rodada
Você modificaria alguma informação no gráfico?	Não	7 (58,3)	4 (50)
	Melhorar destaque da dose, e melhorar distribuição/posicionamento do tempo de infusão e *clearence *de creatinina	2 (16,6)	-
	Melhorar legendas dos gráficos (devem ser autoexplicativos)	1 (8,3)	1 (12,5)
Você teria mais alguma sugestão ou crítica para o aprimoramento do aplicativo?	Considerar presença de mais comorbidades	1 (8,3)	1 (12,5)
	Ajustar e centralizar o layout do software	2 (16,6)	-
	Esclarecer qual CIM a ser considerado	2 (16,6)	2 (25)
	Corrigir o ponto final e a vírgula, pois geram resultados divergentes ao serem inseridos	1	-
Alguma informação é duvidosa?	O valor da CIM a ser considerado não é claro	2	1 (definir CIM e qual é seu significado clínico)
	Entrada inicial “more”. Entrada inicial do software é duvidosa.	3	-

Resultados expressos como n (%). CIM: concentração inibitória mínima.

Foram realizadas mudanças no layout do software antes e após as sugestões dos juízes, não tendo sido registradas dificuldades quanto ao uso do software. Ele foi, então, implementado na rotina da UTI, para realização da segunda etapa da pesquisa.

Na segunda etapa, foram admitidos na UTI, no período de recrutamento, 29 pacientes com indicação de uso de piperacilina/tazobactam. Destes, 21 preenchiam todos os requisitos para inclusão no estudo, sendo 20 pacientes do primeiro bloco de randomização e um do segundo bloco.

Os grupos foram compostos por pacientes predominantemente do sexo masculino (66,6%) com idade variando entre 48 e 87 anos e mediana entre os grupos de 70,14 anos para o grupo dose ajustada e 67,0 anos para o grupo dose padronizada. A tabela 4 apresenta as características antropométricas dos grupos. Não houve diferença estatisticamente significativa entre eles em nenhuma das variáveis sociodemográficas avaliadas neste estudo (p>0,05).

**Tabela 4:** Características antropométricas dos grupos dose padronizada e dose ajustada

Característica	Grupo Dose Ajustada (n=9)	Grupo Dose Padronizada (n=12)	Valor de p
Idade, anos	70,14 (48-87)	67,0 (57,0-79,0)	0,367^*^
Sexo			
Feminino	4 (44,4)	3 (25,0)	0,397^†^
Masculino	5 (55,6)	9 (75,0)	
Altura, metros	1,63 (1,57-1,67)	1,62 (1,56-1,68)	0,968^*^
Peso, kg	65,2 (58,6-97,6)	75,2 (60,9-92,3)	0,604^*^

Resultados expressos como mediana e intervalo interquartil ou n (%).

^*^ Teste de Mann-Whitney; ^†^ teste exato de Fisher.

Os resultados das culturas de material biológico foram disponibilizados para 18 (86%) pacientes. Não houve crescimento detectado de bactérias em culturas de nove pacientes (43%), e, para os pacientes com resultados apresentando crescimento bacteriano, oito (38%) apresentaram perfil de suscetibilidade sensível e um (4,8%) intermediário a piperacilina/tazobactam. Para os demais pacientes, não foram solicitadas culturas (3; 14,3%).

Não se verificaram diferenças para as doses totais administradas entre os grupos. Por outro lado, tanto a dose administrada por horário quanto a frequência de administração destas doses apresentaram diferença entre os grupos (p<0,0001). Em relação ao perfil clínico dos participantes do estudo, não houve diferença em relação às variáveis clínicas e laboratoriais no início da antibioticoterapia (D0), conforme apresentado na tabela 5.

**Tabela 5:** Características clínicas, laboratoriais e dose administrada de piperacilina/tazobactam entre os Grupos Dose Padronizada e Ajustada no início da antibioticoterapia (D0)

Variável	Grupo Dose Ajustada (n=9)	Grupo Dose Padronizada (n=12)	Valor de p
Sepse, n (%)			
Sim	5 (55,6)	8 (66,7)	0,673^*^
Não	4 (44,4)	4 (33,3)	
Uso prévio de antibiótico, n (%)			
Sim	8 (88,9)	8 (66,7)	0,338^*^
Não	1 (11,1)	4 (33,3)	
Foco infeccioso			
Pneumonia adquirida na comunidade	3 (33,3)	2 (16,7)	0,969^†^
Pneumonia relacionada à assistência à saúde, n (%)	5 (55,6)	8 (66,6)	
Outros, n (%)	1 (11,1)	2 (16,7)	
SAPS 3	63,0 (51,0-69,5)	51,5 (43,3-63,5)	0,193^‡^
SOFA	5,0 (4,5-6,0)	5,5 (3,3-7,0)	0,862^‡^
MODS	3,0 (1,5-3,5)	2,5 (2,0-4,0)	0,862^‡^
Dose diária, g	9,45 (9,0-19,63)	13,5	0,4221^‡^
Frequência de administração, n (%)			
3 vezes (a cada 8 horas)	0 (0)	12 (100)	<0,0001
4 vezes (a cada 6 horas)	5 (55,6)	0 (0)	
6 vezes (a cada 4 horas)	4 (44,9)	0 (0)	
Dose administrada no intervalo de dose, g	2,25 (2,25-3,28)	4,5	<0,0001
Creatinina sérica, mg/dL	0,9 (0,6-1,1)	1,1 (0,9-1,6)	0,129

Resultados expressos por mediana e intervalo interquartil ou n (%).

^*^ Teste exato de Fisher; ^†^ teste χ^2^ de Pearson; ^‡^ teste de Mann-Whitney.

SAPS 3: *Simplified Acute Physiology Score*; SOFA:* Sequential Organ Failure Assessment*; MODS: *Multiple Organ Dysfunction Score.*

Ao avaliar os escores e os desfechos, observou-se ausência de diferenças estatisticamente significativas, exceto MODS no quinto dia de antibioticoterapia (D5). Destaca-se que, apesar do grupo dose padronizada possuir menores valores do MODS no D5, esta diferença não se manteve no decorrer do tratamento. O cálculo do SAPS 3 no 28º dia não foi possível, pois eram poucos os pacientes para análise (Tabela 6).

**Tabela 6:** Desfechos clínicos e laboratoriais dos Grupos Dose Padronizada e Dose Ajustada durante o tratamento (D3, D14, D30)

Variável	Grupo Dose Ajustada (n=9)	Grupo Dose Padronizada (n=12)	Valor de p
Tempo de UTI, dias	17,0 (14,0-22,0)	8,5 (6,3-17,5)	0,219^*^
Dias de UTI, n (%)			
≤14	1 (11,1)	5 (41,7)	0,148^†^
>14	8 (88,9)	7 (58,3)	0,382^†^
Dias de antibiótico	7,0 (7,0-10,0)	6,3 (6,0-7,3)	0,277
Creatinina sérica no D3, mg/dL	0,9 (0,6-1,3)	0,9 (0,8-1,5)	0,837^*^
SOFA			
D0	5,2 (3-8)	5,4 (1-9)	0,382^*^
D5	6,0 (3,5-6,0)	3,5 (1,0-6,8)	0,601^*^
D7	3,5 (1,8-6,3)	4,0 (3,0-6,0)	1,0^*^
D14	3,5 (1,8-4,8)	3,0 (2,0-4,5)	0,827^*^
MODS			
D0	2,6 (0-4)	2,8 (1-5)	0,049^*^
D5	3,0 (1,5-4,5)	1,0 (0,3-2,8)	0,669^*^
D7	1,5 (0,8-5,0)	2,0 (1,5-4,0)	0,329^*^
D14	1,5 (0,8-3,0)	3,0 (1,5-3,0)	0,382^†^
Evolução^‡^			
Término do tratamento, n (%)	6 (75)	5 (41,7)	0,197^†^
Óbito, n (%)	2 (25)	7 (58,3)	

Resultados expressos por mediana e intervalo interquartil e n (%).

* Teste de Mann-Whitney; ^†^ teste exato de Fisher; ^‡^ n=20; um dos participantes apresentou resultado de cultura com resistência intermediária a piperacilina + tazobactam, sendo seu tratamento antibiótico modificado.

UTI: unidade de tratamento intensivo; SOFA:* Sequential Organ Failure Assessment*; MODS: *Multiple Organ Dysfunction* Score.

Houve boa aceitação da equipe de prescritores em relação ao uso do software. Não foram registrados eventos adversos relacionado ao uso de piperacilina/tazobactam, mas houve vários registros da equipe técnica de enfermagem em relação às dificuldades encontradas na administração de piperacilina/tazobactam durante o período de estudo.

## DISCUSSÃO

O uso de um software de ajuste e individualização de dose de antibióticos pode contribuir para o uso racional de antibióticos, otimizando e maximizando sua eficácia terapêutica.^6^ Por outro lado, sua utilização em idioma estrangeiro, sem a adaptação linguística e cultural para a língua portuguesa, pode desencadear erros de entendimento e provocar contradições.^7^ Desta forma, sua adequação e validação são maneiras de evitar eventuais erros ou equívocos relacionados à ambientação linguística, como verificado no presente estudo – por exemplo, ajustes de idioma, como a substituição do ponto final pela vírgula, foram necessários, para que não fossem ocasionadas divergências nos cálculos do programa computacional.

Houve concordância superior a 80% em todos os itens avaliados já na primeira rodada, e, após a realização das adequações, na segunda rodada, observou-se aumento na concordância. O alcance de uma concordância superior a 90% em todas as perguntas denotou homogeneidade na avaliação dos juízes, apesar das diferentes áreas de atuação e de profissões.

Destaca-se, ainda, a sugestão de alguns juízes que solicitaram uma melhor caracterização da CIM, a qual tem grande importância no controle das infecções bacterianas. O entendimento da CIM e de suas consequências é de fundamental importância para uma ótima utilização do software.

Estudo recente, avaliando a capacidade de atingir o alvo terapêutico em pacientes sépticos e criticamente doentes, analisou dados de 68 UTI em 10 países e verificou que a dose padronizada, associada à frequência de administração habitual, não atingiu concentrações que efetivamente fossem capazes de abranger todos os organismos suscetíveis.^12^ Em outros estudos, a dose ajustada aumentou a probabilidade de se atingirem os alvos terapêuticos preconizados, principalmente em subpopulações de pacientes criticamente doentes.^5^ O presente estudo evidenciou que, utilizando o software para ajuste individualizado, e com o alvo de CIM em 8mg/dL, as doses totais diárias foram estatisticamente similares às do grupo dose padronizada. Apesar de terem sido fornecidas doses totais similares, o software otimiza a forma de administração do fármaco, ocasionando um fracionamento da dose e aumentando sua frequência de administração.

Vários estudos têm proposto que antimicrobianos administrados por infusão contínua ou prolongada apresentam taxas de cura clínica superiores, quando comparados à administração por dose convencional, e que a individualização de dose, baseada na estimativa bayesiana e em covariáveis clínicas, apresenta maior probabilidade de atingir concentrações terapêuticas capazes de eliminar a infecção e reduzir o surgimento de resistência.^13-16^ Evidencia-se, no presente estudo, a tendência de menor mortalidade no grupo dose ajustada. Mesmo no quinto dia, no qual foram encontrados escores de gravidade mais elevados no grupo que teve a dose ajustada em comparação com os do grupo dose padronizada, observou-se um desfecho similar e numericamente inferior em relação ao total de mortes no decorrer do estudo. O baixo número de participantes pode ter influenciado no desfecho, impedindo extrapolações, mas, ainda assim, nossos resultados permitem inferir a possibilidade de se utilizarem os softwares de ajuste de dose desenvolvidos com base populacional estrangeira na população brasileira.

Muitas vezes, doses elevadas são necessárias para se atingirem concentrações adequada, para tratar infecções causadas por organismos menos suscetíveis (por exemplo, *Pseudomonas aeruginosa* e *Acinetobacter baumannii*).^17^ Além disso, para antibióticos tempo-dependente, quando se aumenta a frequência de administração, ou seja, reduz-se o intervalo entre as doses, a probabilidade de alcance do alvo PK/PD aumenta consideravelmente, uma vez que se reduz também o tempo em que o antimicrobiano necessita ficar acima da CIM. Consequentemente, o aumento da percentagem do tempo acima da CIM (%T≥CIM) está relacionado a resultados clínicos mais favoráveis.^6,18,19^

Uma consideração importante é, que no presente estudo, foi evidenciada a dificuldade de adaptação da equipe em realizar o fracionamento, o armazenamento e a administração da piperacilina/tazobactam em doses fracionadas e em intervalos diferentes dos habituais na dose padronizada. Erros na administração podem responder por 13% de eventos adversos em UTI. Ainda, observam-se taxas de eventos adversos medicamentosos potencialmente evitáveis duas vezes superior em UTI, em comparação com unidades não intensivas.^20,21^ Desta forma, instituições que optam por utilizar dose ajustada precisam investir em treinamento da equipe e criar barreiras de segurança para evitar tais erros.^22^

Dentre as limitações do presente estudo, verificou-se o baixo índice de respostas dos participantes na primeira rodada. Não existe um consenso determinando o número ideal de participantes para realização da técnica de Delphi. Reid^23^observou variação de 10 a 1.685 participantes entre os estudos. No presente estudo, obteve-se taxa de retorno de 10%, ou seja, valor abaixo dos encontrados em inquéritos online realizados na América do Norte, Europa e Oceania, que tiveram média de 33%.^24^ Estratégias descritas para aumentar a taxa de respostas, como reenvio por três vezes do questionário e envio em horários comerciais, foram utilizadas no presente estudo. Alguns fatores podem ter influenciado este resultado: os participantes foram convidados apenas por meio da carta convite por e-mail, sem prévia abordagem pessoal dos pesquisadores; não houve garantia de leitura do e-mail; e muitos e-mails podem ter caído automaticamente na lista de spams. Habitualmente, para as análises estatísticas, o número de participantes é determinado após o aceite em participar da técnica Delphi, sendo contabilizadas as abstenções apenas entre que aceitaram responder o questionário, mas não responderam. No nosso estudo, todos os juízes convidados cujos e-mails não retornaram foram contabilizados para definição da taxa de resposta, o que pode explicar a divergência com a literatura na primeira rodada. A segunda rodada, por sua vez, apresentou retorno de 72,7%, que é compatível com a literatura.^25^

A utilização do software traduzido e adaptado para o português e para a realidade brasileira transcorreu sem dificuldades. Em relação ao tratamento dos pacientes com doses ajustada e padronizadas, não foram observadas diferenças estatísticas entre os grupos, mas são necessários mais ensaios clínicos randomizados, para avaliar desfechos clínicos entre as doses e a verificação de suas concentrações séricas.

## CONCLUSÃO

O presente estudo evidenciou que a versão do software em português é semanticamente segura e apresenta bom nível de validade, permitindo sua utilização no Brasil com confiabilidade, no que concerne à adaptação linguística e cultural. Além disso, a realização do piloto permitiu realizar os ajustes para melhorar a adaptação cultural do software* Individually Designed Optimum Dosing Strategies* e, com base nos resultados obtidos, concluímos que é viável sua utilização em pacientes críticos no Brasil. Deve-se dar continuidade nas avaliações e nos ajustes do programa computacional, até se obterem amostras de concentrações plasmáticas de base populacional nativa. Outros pontos importantes a serem considerados são o treinamento e a orientação da equipe da unidade, para o ajuste e a administração adequada das doses ajustadas.
